# Therapist experiences with implementation of blended (iCBT and face-to-face) treatment of alcohol use disorder (Blend-A): mixed methods study

**DOI:** 10.3389/fdgth.2025.1429582

**Published:** 2025-06-17

**Authors:** Kristine Tarp, Regina Christiansen, Randi Bilberg, Caroline Dalsgaard, Simone Borkner, Marie Folker, Anette S. Nielsen

**Affiliations:** ^1^ National Research Centre for the Working Environment, Copenhagen, Denmark; ^2^ Department of Clinical Research, Research Unit of Digital Psychiatry, Faculty of Health Sciences, University of Southern Denmark, Odense, Denmark; ^3^ Centre for Digital Psychiatry, Mental Health Services in the Region of Southern Denmark, Odense, Denmark; ^4^ Department of Clinical Research, Unit of Clinical Alcohol Research, Faculty of Health Sciences, University of Southern Denmark, Odense, Denmark; ^5^ Psychiatric University Hospital, University Function, Region of Southern Denmark, Odense, Denmark; ^6^ Department for Finance and Planning, Lillebaelt Hospital, University Hospital of Southern Denmark, Vejle, Denmark; ^7^ Department of Clinical Research, Brain Research Inter-Disciplinary Guided Excellence, Faculty of Health Sciences, University of Southern Denmark, Odense, Denmark

**Keywords:** internet-based, alcohol use disorder treatment, mixed methods, blended treatment, therapist perspective, implementation, normalization process theory

## Abstract

**Introduction:**

Though therapists' experiences of offering internet-based treatment for alcohol use disorder have been examined in previous studies, the process of implementing blended internet-based and face-to-face treatment has so far not been studied. This study aims to investigate therapist experiences during implementation of blended face-to-face and internet-based treatment for alcohol use disorder.

**Methods:**

The study employed a mixed methods design, more specifically a triangulation design with a convergence model. Quantitative data using NoMAD were collected in two waves, involving 48 therapists at the 1st wave and 18 at the 2nd wave. Qualitative interviews were conducted six months after the 2nd wave. Eleven therapists participated in focus group interviews for qualitative data collection, and an additional three semi-structured interviews were recorded, transcribed, and subsequently analyzed using the Normalization Process Theory.

**Results:**

We found that the therapists generally had a positive experience with implementing blended face-to-face and internet-based treatment for alcohol use disorder and that their motivation to implement increased. The therapists found it challenging to find coherence between digital and face-to-face treatment in the beginning of the implementation process; however, later in the process, they experienced sense-making. Furthermore, the therapists reflected on their own practice regarding the intervention, both in terms of the amount of time spent on the platform and how it was received by the patients. Moreover, the therapists perceived that if they had all been engaged in the intervention to begin with, it would have led to a shared understanding of the platform and collective ownership. Finally, through each of their individual experiences, the therapists had gained adequate knowledge of the digital intervention; thus, had come to each of their individual perceptions of the best way to incorporate the digital technology in their workday.

**Discussion:**

Familiarity and perceived normalcy of using Blend-A did not change significantly over time, but the cognitive attitude to Blend-A did. The therapists were optimistic about the possible use of a blended treatment format, and that this had a positive effect on the implementation process. Over time, the therapists developed confidence in benefits and disadvantages of a blended format.

## Introduction

### Background

Offering treatment for Alcohol Use Disorders (AUD), for example cognitive behavioral therapy (CBT), via the internet (iCBT) can be an effective way to overcome barriers towards treatment seeking, such as stigma (1). Research has shown that iCBT can be effective in reducing alcohol intake and improving outcomes for both physical and mental health conditions (2–4). iCBT can be delivered in various forms; from pure self-help interventions consisting of self-guided programs, to therapist-guided online programs comprising components with reading materials, assignments, feedback moments, and digital communication with the therapist, and to blended treatment programs, which combine online therapist-guided sessions with in-person sessions (5).

Therapist-guided internet-based interventions are found to rely heavily on the client's intrinsic motivation, and in-person treatment, and guided internet-based treatment is found to appeal to different groups of patients and therapists (6). Ekström & Johansson (4) found that, from the therapist's perspective, being a good therapist in an online setting requires specific considerations and skills. The therapists in their study considered it harder to establish an alliance with patients who only used digital solutions, with communication in writing with the therapist. According to the therapists, it thus became important to communicate in person with the client between homework assignments to create a more personal contact. While the significance of the therapeutic alliance between practitioner and client in the success of iCBT is well acknowledged, the objective of this study is to examine how the implementation of iCBT manifests in practice.

### Implementation

Though therapists' experiences of offering iCBT for AUD have been examined in previous studies, the process of implementing blended iCBT and face-to-face (FtF) treatment has not so far. Normalization Process Theory (NPT) (7) is frequently used in qualitative research aiming to understand and evaluate the processes that shape the implementation and delivery of healthcare innovations within organizations. The theory is particularly well-fitted in terms of introducing new and complex digital tools in the healthcare system when it comes to the designing of complex interventions and understanding the dynamics of implementation processes and their outcomes.

NPT is a translational framework, based on empirical studies and evidence syntheses. NPT outlines three context-mechanism-outcome (CMO) domains that have the potential to impact implementation: implementation contexts, implementation mechanisms, and implementation outcomes. All three domains build on primary constructs; and in addition, the second domain, implementation mechanisms, also comprises sub-themes. In present study, we focus on the four primary constructs within the domain implementation mechanisms. May et al. (7) define mechanisms as the work people do when they participate in implementation processes. The domain includes the constructs coherence building, cognitive participation, collective action, and reflexive monitoring, elaborated on below. All constructs can be measured by the means of quantitative data. However, while the constructs all are of importance, the therapists may have additional or alternative views and reflections that questionnaires and quantitative measures may not bring to light. Uncovering this information needs alternative qualitative research methods.

The construct coherence building refers to people's sense-making of an intervention by means of the way they work together in everyday settings to understand and plan the activities, which need to be accomplished to put the intervention and its components into practice. Coherence building comprises four sub-themes, namely differentiation, communal specification, individual specification, and internalization. The sub-themes each respectively represents modes of sense-making internal and external to the involved individuals in terms of understanding various components of the intervention and overcome difficulties associated with the implementation. E.g., the sub-theme differentiation concerns people's ability to understand a set of practices and their objects, which may be understood differently.

The construct cognitive participation comprises the sub-themes initiation, enrolment, legitimation, and activation. These sub-themes cover aspects of practices, which are new or perhaps need to be modified, where a core challenge may be whether key participants are able or willing to drive the practices forward. In the present context, the therapists must organize or reorganize themselves and others to collectively contribute to the work involved in the new practice. This is a complex working process that may involve rethinking individual and group relationships between people and things.

The construct collective action concerns the ability to work together to endorse an intervention and its components. This may, e.g., refer to the allocation of work, which underpins the division of labor that is built around a set of practices as they are operationalized in the real world.

The construct of reflexive monitoring comprises systematization, individual appraisal, reconfiguration, and communal appraisal, which refers to collecting information, expressions of personal relationship to new technologies, redefinition of procedures, modifying practices, or evaluating the worth of a set of practices. Participants in any set of practices may seek to determine how effective and useful it is for them and others, and this involves the work of collecting information in multiple ways.

A series of phenomena are in play during implementation processes, when therapists are about to adopt a new treatment offer, embedding and routinizing new ways of working. When examining these phenomena, the NPT is a relevant theoretical framework to use, since it involves identifying, differentiating, and codifying the qualities and characteristics of such phenomena. Thus, NPT provides the mechanisms to examine how and why cognitive and social processes are critical for implementation and explains how changes occur in the way employees use and consider an innovation (8).

### Aim

Using mixed methods, the present study examines how therapists engage into the process of implementing the *Blending Internet treatment into conventional face-to-face treatment for alcohol use disorder* (Blend-A) (9) treatment platform. The implementation process is viewed through the lens of quantitative and qualitative data material. The quantitative data are extracted from The Normalization MeAsure Development questionnaire (NoMAD) (10–12). The therapists' clinical experiences of transitioning from FtF to blended treatment are uncovered through qualitative interviews and later analyzed by the means of NPT mechanisms.

## Materials and methods

### Design

This study is conducted in a mixed methods design, using triangulation and the convergence model (13). Survey data using NoMAD were collected in two waves. Qualitative interviews were conducted six months after the 2nd wave. By using this model, quantitative and qualitative data were collected and analyzed separately and subsequently the results were compared. The purpose of using this model was to formulate valid and well-substantiated conclusions about the therapists' perspective of implementation of Blend-A. The consolidated criteria for reporting qualitative studies (COREQ) (14) were used as a checklist for reporting.

### Setting

In Denmark, treatment for alcohol use disorder (AUD) is provided by municipalities and is free of charge for the patients. Patients have the option to choose which municipality they wish to receive treatment in and are guaranteed treatment within 14 days. Treatment typically consists of conversational therapy, including Motivational Interviewing (MI), Cognitive Behavioral Therapy (CBT), supportive conversations, family therapy, and network-reinforcing initiatives, and may also include pharmacological treatment. The most common forms of treatment for AUD are MI and CBT (15, 16). Treatment duration is typically four to six months, depending on the patient's needs, and is provided by therapists with professional backgrounds as nurses, psychologists, pedagogues, and social workers.

### Context

This study is a sub-study of the larger trial Blend-A (9) and investigates the implementation process of Blend-A in alcohol treatment institutions, located in various municipalities. Blend-A is a treatment program aimed at treating AUD by combining the use of an online digital treatment platform with FtF-sessions at municipal alcohol treatment institutions. This mode of treatment originated in the Netherlands and has been found successful in the implementation of AUD treatment programs (17). The online treatment platform is flexible, the patient can choose to not store personally attributably data on the platform and thus stay anonymous to the system except to the therapist. The platform consists of 21 modules, which patients can choose from in collaboration with the therapists, and the patients can review previous answers and receive feedback from therapists on current issues (18).

All Danish Municipal treatment institutions for AUD were offered participation in the overall Blend-A trial. The trial was designed as a pragmatic stepped wedge cluster randomized controlled study of outcome of Blend-A (9), and 18 institutions accepted the invitation. The implementation of Blend-A was facilitated by members of the author group (RB, KT, and ASN) in the treatment institutions in random sequences between June 2020 and December 2022. Four institutions dropped out during the study period. During the process of implementation, RB, KT, and ASN offered training and supervision aimed at supporting the therapists' ability to use the Blend-A treatment form and had therefore to some extend established some kind of professional relationships with the therapists prior to study commencement.

### Data collection

Quantitative data consisted of data collected through a survey in two waves. The survey was sent to all employees at the 14 remaining alcohol treatment institutions via their work e-mail. The survey comprised questions covering demographic information and the implementation process, including the NoMAD questionnaire. NoMAD is based on NPT and was used to study the implementation process. NoMAD operationalizes the implementation process through the four primary NPT-constructs within the CMO-domain “implementation mechanisms”: *coherence, cognitive participation, collective action, and reflexive monitoring* (see Table 1). The questionnaire consisted of three general questions and is followed by 20 questions on a Likert scale to measure the employees’ familiarity with the implementation process. Prior to answering, the participants were informed of the purpose of the study. Data was collected in two waves with five months in between. The questionnaires were filled out through the system Research Electronic Data Capture (REDCap) in Open Patient data Exploratory Network, OPEN.

**Table 1:** NPT CMO-domains, primary constructs and sub-themes.

CMO-domains	Primary constructs	Sub-themes
Implementation contexts	Strategic intentions *How do contexts shape the formulation and planning of interventions and their components?*	
	Adaptive execution *How do contexts affect the ways in which users can find and enact workarounds that make an intervention and its components a workable proposition in practice?*	
	Negotiations capacity *How do contexts affect the extent that an intervention and its components can fit, or be integrated, into existing ways of working by their users?*	
	Reframing organizational logics *How do existing social structural and social cognitive resources shape the implementation environment?*	
Implementation mechanisms	Coherence building *How do people work together in everyday setting to understand and plan the activities that need to be accomplished to put an intervention and its components into practice?*	Differentiation Communal specification Individual specification Internalization
	Cognitive participation *How do people work together to create networks of participation and communities of practice around interventions and their components?*	Initiation Enrolment Legitimation Activation
	Collective action *How do people work together to enact interventions and their components?*	Interactional workability Relational Integration Skill-set workability Contextual Integration
	Reflexive monitoring *How do people work together to appraise interventions and their components?*	Systematization Communal appraisal Individual appraisal Reconfiguration
Implementation outcomes	Intervention performance *What practices have changed as the result of interventions and their components being operationalized, enacted, and reproduced, over time and across settings?*	
	Relational restructuring *How have working with interventions and their components changed the ways people are organized and relate to each other?*	
	Normative restructuring *How have working with interventions and their components changed the norms, rules and resources that govern action?*	
	Sustainment (normalization) *How have interventions and their components become incorporated in practice?*	

The qualitative data for analyzing the implementation process of Blend-A was collected through three semi-structured focus group interviews and one individual interview using an NPT-based interview guide (Supplementary file). The interview guide asked open-ended questions to the therapists about their workday with Blend-A, how they used Blend-A in their institutions, and how they used Blend-A in their treatment courses. The four interviews were conducted by RB, RC, and KT via videoconferencing in Cisco Webex. The interviews lasted between 34 min and 76 min and were all audio recorded and transcribed by author SB. No observers were present during the interviews and no field notes were taken.

### Data analysis

Data from the questionnaires was analyzed using descriptive statistics. To assess differences in NoMAD questionnaire responses between the 1st and the 2nd wave, a Wilcoxon rank-sum test was employed since the data did not follow a normal distribution. All statistical analyses were conducted using STATA Software Package 17.0 (StataCorp., 2022).

The transcribed interviews were analyzed in NVivo using the General Inductive Approach (GIA) (14). This specific approach is often used when analyzing qualitative evaluation data. The main purposes of the approach is to (a) condense raw data into summary format (b) establish a clear link between the research objective and the findings from the raw data and, (c) create a process-oriented framework on the basis of the structure of experiences/perceptions from the raw data. GIA allows the inductive data analysis to be guided by the aforementioned constructs.

Two coders, authors RC and KT (both female postdocs, who holds MA degrees within anthropology and philosophy, PhD degrees within health sciences, and approximately ten years of experience within qualitative research) independently coded all interview transcripts according to the four primary constructs within the CMO-domain “implementation mechanisms”: coherence building, cognitive participation, collective action, and reflexive monitoring. They then collaboratively compared and discussed their coding. In instances of disagreement or ambiguity concerning the alignment between constructs and data, a third researcher ANS was consulted to facilitate consensus. In the final stage of this process, the remaining co-authors reviewed the constructs alongside the corresponding quotations. Essential quotations were translated from Danish to English by author CHD and included. The final coding decisions regarding the placement of quotations were then made jointly by KT and RC.

## Results

### Participant sample description

In the middle of the study period for the overall Blend-A study, a survey questionnaire was sent to all staff (*N* = 82) in the 14 alcohol treatment facilities to assess the implementation of the Blend-A platform. Forty-eight participants responded, equivalent to a response rate of 58.5% (see Table 2). The response group had a mean age of 51 (SD 10) years and 17% of them were men. The three largest professional groups were social workers (38%), pedagogues (27%), and nurses (25%). The response group had worked an average of 8 (SD 6) years in their current institutions, and they had an average experience of 9 (SD 7) years treating people with alcohol problems. Of those who answered the questionnaire, it was mainly employees (94%) rather than managers, and 81% of the employees used Blend-A in their daily work (See Table 2).

**Table 2:** Information about the participants (*N* = 48).

Categorical variables	%
Sex	
Men	17.0%
Professional groups	
Social worker	37.5%
Nurse	25.0%
Psychologist	2.0%
Pedagogue	27.0%
Other	8.5%
Use of Blend-A in daily work	
No	17.0%
Yes	81.0%
Do not know	2.0%
Function/job description	
Employee	94.0%
Manager	2.0%
Project manager	4.0%
Numeric variables	Mean (SD)
Number of years in the current institution	7.6 (6.3)
Age	50.6 (10.1)
Years of experience with alcohol treatment	9.2 (6.9)

In addition to filling in the questionnaire, all therapists from the participating treatment centers were invited to participate in a focus group interview. In the invitations, the therapists were informed of the purpose of the study. Of these, 11 therapists agreed to participate in four group interviews; however, one interview ended up being individual. We did not ask for reasons from those who did not opt in. The therapists were from seven different municipalities in Denmark. The therapist interview group consisted of nine women and two men. The therapists had a mean age of 40 years (SD 6, range 31–55). The therapists had different educations: five were social workers, three were nurses, one a psychologist, one a pedagogue, and one had put “other” under education. All therapists had used the internet-based alcohol treatment program with patients, except for one, who was an implementation project manager.

### Survey

The results of the scores on the NoMAD instrument from both 1st and 2nd wave as well as the sub-scores are presented in Table 3. It should be noted that the first three questions were rated on a 1–10 scale and were not included in the total score. A positive trend from 1st to 2nd wave is seen; however, not significant.

**Table 3:** 1st wave (*n* = 48) and 2nd wave (*n* = 18) answers to the NoMAD instrument (10–12), total score and subcategories.

NoMAD questions	1st wave Mean (SD)	2nd wave Mean (SD)	*p*-values*
(1) When you use Blend-A, how familiar does it feel? (score 0–10)	4.4 (3.5)	6.6 (2.3)	0.164
(2) Do you feel Blend-A is currently a normal part of your work? (score 0–10)	3.6 (3.2)	5.4 (2.6)	0.151
(3) Do you feel Blend-A will become a normal part of your work? (score 0–10)	5.6 (2.9)	6.1 (2.7)	0.558
(4) I can see how Blend-A differs from usual ways of working (score 0–4)	2.7 (0.9)	2.8 (0.9)	0.301
(5) Staff in this organization have a shared understanding of the purpose of Blend-A (score 0–4)	2.8 (1.1)	2.9 (0.9)	0.730
(6) I understand how Blend-A affects the nature of my own work (score 0–4)	2.4 (1.1)	2.5 (1.0)	0.809
(7) I can see the potential value of Blend-A for my work (score 0–4)	3.2 (0.9)	3.2 (1.1)	0.219
(8) There are key people who drive Blend-A forward and get others involved (score 0–4)	2.8 (1.3)	2.9 (1.4)	0.156
(9) I believe that participating in Blend-A is a legitimate part of my role (score 0–4)	3.1 (0.9)	3.3 (1.0)	0.563
(10) I’m open to working with colleagues in new ways to use Blend-A (score 0–4)	3.5 (0.8)	3.8 (0.9)	0.999
(11) I will continue to support Blend-A (score 0–4)	3.5 (0.8)	3.5 (0.9)	0.250
(12) I can easily integrate Blend-A into my existing work (score 0–4)	2.6 (1.0)	2.7 (1.4)	0.480
(13) Blend-A disrupts working relationships (score 0–4)	1.2 (1.1)	1.1 (1.2)	0.488
(14) I have confidence in other people's ability to use Blend-A (score 0–4)	3.4 (0.9)	3.7 (0.7)	0.500
(15) Work is assigned to those with skills appropriate to Blend-A (score 0–4)	3.4 (0.9)	2.1 (1.6)	0.371
(16) Sufficient training is provided to enable staff to implement Blend-A (score 0–4)	2.0 (1.5)	3.3 (1.0)	0.808
(17) Sufficient resources are available to support Blend-A (score 0–4)	2.8 (1.1)	3.2 (1.0)	0.500
(18) Management adequately supports Blend-A (score 0–4)	3.3 (1.1)	3.1 (1.0)	0.529
(19) I am aware of reports about the effects of Blend-A (score 0–4)	2.1 (1.1)	1.9 (1.4)	0.501
(20) The staff agree that Blend-A is worthwhile (score 0–4)	2.4 (0.9)	2.1 (1.3)	0.918
(21) I value the effects that Blend-A has had on my work (score 0–4)	2.3 (1.0)	2.5 (1.2)	0.999
(22) Feedback about Blend-A can be used to improve it in the future (score 0–4)	3.2 (0.8)	3.6 (0.6)	0.625
(23) I can modify how I work with Blend-A (score 0–4)	3.2 (0.7)	3.3 (0.8)	0.531
Total NoMAD-score and sub-scores			
NoMAD-score (score 0–80) (questions 4–23)	55.1 (9.3)	57.6 (8.8)	0.240
Sub-score, *coherence building* (score 0–16) (questions 4–7)	11.1 (2.5)	11.5 (2.6)	0.524
Sub-score, *cognitive participation* (Score 0–16) (questions 8–11)	12.9 (2.7)	13.4 (2.8)	0.012*
Sub-score, *collective action* (score 0–28) (questions 12–18)	18.0 (3.6)	19.2 (3.2)	0.267
Sub-score, *reflexive monitoring* (score 0–20) (questions 19–23)	13.0 (2.8)	13.4 (3.1)	0.982

* Tested via the Wilcoxon rank sum test.

Scored from 0 to 4, where 0 = disagree and 4 = agree.

We have replaced [the intervention] with “Blend-A”.

Questions four to 23 received an average score of more than two, except for question 13, which concerned the interference of Blend-A with the employees' everyday lives, at both waves. In terms of the sub-scores, there was a significant increase in *cognitive participation* from a score of 12.9 (SD 2.7) at 1st wave to 13.4 (SD 2.8) at 2nd wave (*p* = 0.012). There was a positive trend from 1st to 2nd wave, but no significant changes in the other three scores or in the total NoMAD-score.

### Therapist experiences with implementation mechanisms

In the present section, the qualitative data is used to uncover the therapists' thoughts and experiences involving the mechanisms influencing the implementation process. The results are grouped along the four primary constructs: *coherence building*, *cognitive participation*, *collective action*, and *reflexive monitoring*, thus allowing for an expanded understanding.

#### Coherence building

The therapists described how they struggled to find coherence between the new digital intervention and their well-established experiences with the ordinary treatment form of FtF. More specifically, in the early days of the implementation process, the therapists were concerned about how written communication with and feedback to the patient would function, as it was perceived an opposite to their normal way of communicating verbal and FtF feedback to the patient. Not being able to directly experience the patient's facial expression when communicating was considered a real obstacle to having a meaningful communication and thus for the therapist to deliver an appropriate intervention, adjusted to the patient's needs. This worry was central to the therapist, since being able to be adjusting to patients' needs was considered as being central for creating a solid working alliance with the patients.

However, over time and getting familiar with the program, the therapists slowly underwent a process of sense-making, referred to under the sub-theme communal specification. When allowed time to engage in and explore the intervention, the therapists came to rethink the possible benefits of using the blended format. To the extent that the therapists engaged themselves into the intervention, they expressed how they found the intervention a usable tool in their everyday practice. By this recognition, they came to understand and point to differences between online treatment and FtF treatment. A therapist described how there were multiple modes of using the blended format, and pointed to one specific benefit of the blended format that added to her sense-making of the intervention by reference to collaborating with the patient when shifting between FtF conversations and written digital feedback to the patient on his/her work with the modules:

“.. they shouldn't think of the conversation as just another module, but maybe think that the conversation is.. well, more like, is there something to pick up on from what you've already been through? Or if you feel, ‘Hey, there’s actually a theme here that you mentioned in that session, where I didn’t provide feedback. Would it make sense to you if you come in, and we could work on that a bit?’ And then you can proceed with the remaining modules.” (Therapist 1)

Here we find sense-making internalized through each patient's individual need for feedback. In coherence with the patients, the therapists sought to work more thoroughly with the intervention by keeping an eye on alternative ways to interact with the patient during the treatment. By the blended intervention the therapist gained new understandings about treatment and responsibilities to engage with the patient.

One therapist mentioned how recognizing the use of the platform as an instrument for solving immediate questions that may arise, changed his/her perceptions of the possibilities inherited in the digital platform—possibilities, which differentiated from the more familiar therapeutic FtF sessions. By the therapists becoming increasingly aware of the difference of the two therapeutical options, they came to gain more visibility about own resources available for working with the digital intervention on a long-term perspective. A therapist explained how the digital intervention could be integrated in or change already existing routines:

“Yes, and the patient also gets the experience of ‘well, if there’s something I'm in doubt about, then I'll just write’, ‘well go ahead and do that’ and I could also see when messages ticked in: ‘Now there’s actually something on the platform’. And I could see, it’s a conversation ‘Well, I'll just take this right here between two sessions’ because it’s like an SMS, you don't have to spend a lot of effort on that. But as the patient also experiences, it sparks a connection.” (Therapist 1)

Sense-making also involves internalization, which concerns the therapist's ability to understand the value, benefits, and importance of a set of practices. It is about being able to attribute worth to a new way of working. According to the therapists, the work of providing feedback was not only considered to be of benefit to the patient but also something, which offered the therapist a room for providing more in-depth therapy. When the therapists had to provide written feedback, they realized that they became more concerned about weighing up his/her feedback based on their best body of knowledge of the specific patient on the one hand and how the patient would receive the feedback on the other hand.

The therapists also realized that the contact to the patient was more dynamic in structure as there might occur communication between therapist and patient through the platform in between FtF-sessions. A therapist explained how the intervention had not really changed the normal workflow, the feedback module had rather added to the therapist's ability to comprehend the patients' unwritten thoughts and perspectives. In that sense, it offered the therapist, unlike in the FtF-sessions, a space for thinking more thoroughly about the patients' reflections during the treatment course.

#### Cognitive participation

When the therapists were asked about their experiences on implementing blended care into their daily practices, they placed emphasis on the importance of planning their weekly schedule as to having time for working with the new online platform. A therapist stressed that using the digital platform required following a structure and integrating the working procedures of the platform into the therapists' calendar and workflow, especially if the therapist is to communicate with several patients. The therapist described:

“… Well, I found it nice that I had a day reserved for it, it gave some peacetime. However, I could also see that if I had gaps here and there, I have never adhered to it so strictly, because I also know that I get cancellations during the week, or some might cancel, and then I naturally use that time to provide feedback. It’s more for the patients’ sake, so that they know that they had something to relate to. That uncertainty, which we all know is a plague ‘when will I get feedback on this?’, ‘Oh, I know I will at least always get it on Tuesdays.’” (Therapist 1)

In making sense of a blended format of treatment, the therapists in general acknowledged ways to overcome the risk of placing unnecessary scruples to oneself. In partnership the therapists build a shared understanding of the best way to overcome such risk, for example as explained by one of the therapists:

“…we quickly agreed that it would make a lot of sense to structure the feedback. So, telling the patients, ‘Well, we provide feedback once a week’, and then you simply allocated an hour. At that time, each of us allocated an hour each week, telling people, ‘Here, you can expect to receive feedback’. Personally, I set aside Tuesday mornings from 8 to 9, informing people that ‘even if you may want to proceed quickly and are very eager, well, you can't expect to get feedback the day after you've sent it. I aim for it, but if I'm busy, you might not get it, but you can at least expect that you'll always get feedback on a Tuesday.’” (Therapist 1)

As to ensure that the platform successfully became a beneficial element in the treatment, the therapists were aware of the importance of securing the right information and encouragement of the patients through profound relational work. This was best done by ensuring that the patients know what the intervention applied to and how the intervention could be of benefit to the individual patient.

By getting to know the digital intervention and its possibilities and limitations, the therapists became enabled to reflect on their own practice regarding the intervention, both in terms of the amount of time spent on the platform and how the platform was received by the patients. By acknowledging various aspects of difficulties or limitations connected to the use of the platform, it was considered necessary to adjust the intervention to make it more accurately fit the patient's needs and capabilities in using the treatment program. In one treatment center, the therapist had the following way to overcome hurdles associated with the intervention performance:

“Well, we've had a facilitator, who has been responsible for conveying information regarding you (the provider of Blend-A) and has participated in those meetings until we decided that we wouldn't invest more effort in it. And then all the therapists in the team, the six therapists, have had access to it, and everyone has had the opportunity to offer it to their patients. Occasionally, we've had some theme days where we've considered how we could use it more creatively and in a way that suits our people. And then I recognize that when you've just brainstormed on it, it comes up more than when you haven't.” (Therapist 2)

#### Collective action

The therapists in general considered that if they had all been engaged in the intervention to begin with, it would have led to a shared understanding of the platform and collective ownership. Primarily because had all therapist's been involved in the intervention, there wouldn't have been a division between those working with the platform and those not working with the platform, which may have led to negative relational integration. In the present context, the relational integration refers to the knowledge work, which the therapists build together to maintain confidence in the digital platform and in the way they use the platform, something that is difficult to succeed with when a shared understanding is not present from the onset. Especially the module of providing the patient feedback occupied the minds of the therapists. Providing the patients with feedback was circumscribed with feelings of being insecure about the therapist's role and skills to perform this task. To both patients and therapists providing online feedback was not part of their usual interaction and communication form, and in addition, they worried about not being able to adjust their communication according to facial expressions. The therapists mentioned various ways they applied to skill set workability:

“Yes, because I can feel that my feedback—now that I know that it is spot on—then I know that I can write in the feedback what they need to think about. And then you can say, you start to get into a routine, so you don't have to think deeply every time. But then you can say, I feel, sometimes Blend-A might be deprioritized if they haven't done something themselves on the day when I give feedback. Then I'm not always that pro at them, because then, ‘oh’, I can feel that I have something else for next week, and then I hope it was a better week where I got, um… And sometimes, a month can actually go by where I think, ‘damn, I should have addressed them earlier’, but yeah, because it becomes theirs. And if they're not quite on it, I can end up stretching it a bit. .. It requires some ownership, and they need to know and commit to that, yeah.” (Therapist 3)

One way to uphold interactional workability was for the therapists to ensure that they aligned their uses of the digital intervention. A therapist mentioned how regular meetings were important in the process of interactional workability:

“…And otherwise, we've have used the meetings, for those of us involved in it and dealing with the feedback in Blend-A, also to align it a bit, how one did it, how one thought, how long a thread do we give people when they are inactive before we say, ‘Now you need to come in for a conversation, and maybe we'll stop it because it doesn't work when you're not doing anything.” (Therapist 4)

Another therapist mentioned how they would have preferred all therapists in the treatment center to be familiar with the digital platform to maintain confidence and build accountability in a set of practices and in each other as they use them:

“Initially, there were only three of us, who were selected to participate [in the Blend-A project] and had learned how to use the platform. We should have included all nine because it has done something for ownership, even though we've tried to bring it up and incorporate it into our treatment conference and such. It hasn't come naturally to the other therapists. So, it’s definitely something we take with us that if we're going to participate in such a project again, everyone accessing the platform and being involved in it should be included.” (Therapist 5)

To make the intervention a part of already established procedures, which are well-known to the therapists requires great focus on the contextual integration, which refers to the resource work related to the new intervention. At one treatment center, they decided to implement in-house workshops focusing on finding alternative ways to use the platform as to make it work more in accordance with the workflow at their treatment center. By allowing themselves to meet and evaluate the platform, they acknowledged that further aspects came to light. Aspects that showed the benefits of rearranging the treatment sessions in the case a session was irrelevant to one specific patient or chancing something in accordance with the patient's individual difficulties.

Being aware of the allocation of resources or procedures, would provide the therapist with confidence in the intervention. It was highlighted by one of the therapists that in general the therapists were positive and engaged towards the intervention.

Further, the therapists were aware of the required amount of structure and systematization in the process of integration of the intervention to keep the new platform in view. The therapists also pointed to how, in one treatment center, having internal meetings and assuring that there is a structure to follow in the daily work was perceived helpful. They recognized the process in connecting the therapists to the platform, which was something that required focus on continuity in information as well as supplementing the initial introduction to the platform with a follow-up session.

#### Reflexive monitoring

The therapists found themselves in a new set of practices, and therefore they also had to work experientially as individuals to appraise the digital interventions effects and the contexts in which they already are a part of. From this experiencing work stem actions through which the therapists expressed their personal relationships to the new digital intervention:

“We are two therapists who are committed to this in my department. Now we are expanding to three therapists. And you can say that the fact that there are only three of us who can drive it also means that we have a lot of experience with it now, and it might not be as challenging to have a Blend-A client because you gain some routine and experience with it. And feedback might not take as long either. But I think it’s nice that we become one more, because sometimes I think it’s been a bit ‘phew’, there have been many. Those who have really been well-assessed, they have truly benefited from it and really gained a lot from the treatment, so there I think it has been really good.” (Therapist 3)

The therapists' engagement led to attempts to redefine some already established procedures or modifying their practices. A therapist explained that after being presented to Blend-A, she/he realized that home assignments were something he/she should initiate more often than she/he had done previously, here expressed:

“I think for us, it has also made us consider that there are alternatives. I may not have been very good at making use of homework myself and perhaps have a respect for what it can do, so I've definitely started thinking about that, even with patients I don't have in Blend-A. I've been focused on giving them homework assignments and saying, ‘There’s also some responsibility at home’, meaning saying, ‘You need to complete this before we meet next time.” (Therapist 6)

Through the therapists' individual experiences, they gained more adequate knowledge encompassing the digital intervention. The therapists might have come to each of their individual perceptions of the best way to not only incorporate the digital technology in their workday, but also on how the FtF treatment can be inspired by tools and strategies, originating from the digital platform.

## Discussion

The aim of present study was to investigate the process of implementation of Blend-A by the means of both quantitative and qualitative data, collected from health- and social professionals. In the following section, the results of the analysis of the quantitative and qualitative data, respectively, will be compared, and discussed.

### Coherence building

When looking at the survey results for the therapists' experienced coherence building both individual and in collaboration, there is a high overall score with no remarkable change between the 1st and the 2nd wave. However, delving into the subgroup questions provides valuable insights, particularly in question four, where therapists at the 2nd wave scored 2.8 (SD 0.9). This score suggests a partial agreement among employees that the implementation of Blend-A differs from their usual work processes. When asked about this in the interview a therapist explained that the intervention had minimal impact on their workflow, which could indicate a positive adaptation to the intervention.

Furthermore, in question seven, therapists scored 3.2 regarding their perception of the potential value in Blend-A. This sentiment aligns with the interview findings, showing consensus on Blend-A being a beneficial tool for patients. The interviews also highlighted unanimous agreement that it was easy for therapists to utilize the time between two sessions to respond to messages from patients.

During the interviews, the therapists described how the digital platform might serve as a path to solving immediate or urgent questions from the patient, which potentially could offer both the patient and the therapist a more natural flow in communications and treatment. This finding is in accordance with the conclusions of Månsson and colleagues (19), who found that digital platforms provided therapists with a greater overview of the treatment and the processes involved in the treatment.

We found this supported in our data by means of the therapists' ability to strengthen the transition between written feedback through the digital platform and the therapeutical sessions. This finding is also supported by Ekström and Johansson (4), who found that therapists expressed advantages of having time to prepare, i.e., think for a while, asking someone for a piece of advice, and not having to deliver an answer immediately. Such findings are part of the construct related to *coherence building*, where we found that at early stages of the implementation, the therapists had difficulties in differentiating iCBT from FtF treatment, making it challenging for them to find coherence between the two modes of intervention. In a similar study, Bengtsson and colleagues found that therapists had expressed that this had made them feel as working all the time (6).

### Cognitive participation

In the four sub-questions for the category *cognitive participation*, the therapists scored 3.5 points (SD 0.8) in 1st wave that they were open to working with Blend-A and that they would continue to support Blend-A. The employees thereby expressed that they partially agreed or totally agreed that they were motivated for Blend-A, expressed that they would continue to support the use of Blend-A and were open to using Blend-A. The employees answered the lowest score in 1st wave about whether there were key people in the workplace who drove the implementation of Blend-A [2.8 points (SD 1.3)]. This may indicate that at the beginning of an implementation process it is important that there is somebody who shows the way for the other employees.

In general, we found that, over time, the benefits of the blended format were apparent to the therapists and added to their perceived quality of working online in partnership with the patient. Békés and colleagues (20) consider that such change may be closely tied to professional self-doubt which is a contributor to therapists' acceptance level and positivity regarding online treatment. We believe that aspects of self-doubt as presented by Békés and colleagues (20) may be an important and even overlooked factor for discussing cognitive participation because self-doubt may be what drives forward the therapists involvement in the implementation process and the finding of alternative paths to engage with the new platform that is more in consonance with each of their individual approaches to treatment. This is also supported by Bengtsson and colleagues, who found that following a program adds to the feeling of safety to the therapist as the treatment follows what the patient has agreed upon and that the therapist does not get stuck in small things not relevant to treatment (6).

### Collective action

The therapists scored low on whether the use of Blend-A interfered with collaborative relationships (question 13). On the one hand, this may indicate that the therapists did not consider Blend-A in their collaborative relationship with patients and colleagues, but on the other hand, it may also indicate that Blend-A was successfully implemented and that they did not consider Blend-A as a disturbance of their relationship with patients and colleagues. To figure out whether it is one or the other, it is relevant to examine the other answers in collective action. Question 16 examines whether the employees felt that everyone involved in Blend-A had received sufficient training in the use of Blend-A, with the therapists scoring 2.0 (SD 1.5). This indicates that there was disagreement about this. A comparison between the scores in question 16 and 13 suggests that employees may not have implemented Blend-A sufficiently, as they have not received adequate training. This lack of training may be a factor in not perceiving Blend-A as a disruptive element in collaboration with patients, as insufficient education appears to be an unresolved issue in their response.

This is also illustrated in the interviews, where a therapist mentioned how they would have preferred that all therapists in the treatment center from the onset were familiarized with the digital platform to maintain confidence and build accountability in a set of practices.

During the interviews, we found that to overcome struggles and work overload to each individual therapist, aspects comprising the construct collective action pointed to great collegial interest in finding solutions for the intervention to benefit them in the implementation process. The therapists arranged collective meetings and workshops where they had the opportunity to discuss work to be done to enable the intervention. Furthermore, we believe that findings related to the construct collective action such as joint meetings, collaboration, and sense of community are important aspects for successful digital intervention implementation processes.

### Reflexive monitoring

In the NoMAD questionnaire, the overall score for *reflexive monitoring* was 13.0 at the 1st wave and 13.4 at the 2nd wave. For the questions for this domain, the therapists scored 2.1 at 1st wave and 1.9 at 2nd wave in question 19 regarding their awareness of the effects of Blend-A. This is a rather low score, which may indicate that the therapists were not aware of or perhaps not truly convinced about the benefits of the use of Blend-A. This outcome was somewhat expected, as there was no initial assumption that therapists would possess detailed knowledge on this matter, as this responsibility was delegated to the authors, facilitating the implementation process. However, lack of knowledge regarding an implementation of a new project can result in bad implementation results (21). Thus, half a year later, when the qualitative interviews were performed, the therapists felt increasingly aware of the effects, which might be due to their own experiences by using Blend-A. Regarding question 22 and 23 concerning how feedback from Blend-A could be used to improve the future and the capability to modify how to work with Blend-A, the therapists scored higher.

During the interviews, the therapists also expressed how they increasingly used knowledge, benefits, and experiences from Blend-A in other settings. Furthermore, the feeling of safety in the treatment form as mentioned above is also considered a part of the construct reflexive monitoring, where we as an example found this to be expressed through increased uses of home assignments in relation to FtF-treatments. We considered if a possible positive outcome of increased use of home assignments could add to levels of increased working alliances with the patient, which furthermore would affect and ease the implementation of iCBT because the therapist would find their effort worthwhile. This would be an important finding since Békés and colleagues found that poor working alliance were equal to less positive attitudes towards online treatment (20). We do not think, however, that our data indicate neither of these two outcomes but instead we found that for the therapist to have the time for working with both in parallel and by time getting to feel that the two modes of treatment complement each rather than exclude one another is of importance when implementing new practices.

### Strengths and limitations

Regarding the survey, we saw a low response rate at the 2nd wave with only 37.5% out of those who responded at the 1st wave. This may explain the lack of significant change from autumn 2021 to spring 2022, and the findings should be interpreted with caution.

The NoMAD tool is a useful tool for assessing implementation processes at both the individual and collective levels, and for identifying inhibitors and promotors of the process. However, it was translated into Danish for use in the present study, and we cannot be completely sure that the meaning of the survey questions is preserved in the translation. Additionally, there are no instructions for how to analyze the results, why different studies analyze the data differently. This can make it difficult to compare results across studies, which have used the NoMAD instrument. Despite these limitations, the NoMAD tool is easy to apply and may provide valuable insights into implementation processes. It is a strength of the study that the implementation process was systematically measured.

Regarding the qualitative interviews, we do not assess the relatively small sample size (*n* = 11) to be a limitation, since Guest et al. (22) argue that the first six interviews are crucial for constructing meaningful themes during an inductive analysis. Their recommendation is based on an experiment they conducted with data saturation and variability. With regard to the facilitation of an interviewer-interviewee alliance and thereby the interview validity, Crouch and McKenzie (23) also argue that a small number of participants can be feasible. Since we used independent parallel coding and codes check, the study was strengthened according to the internal validity and reliability (24), enhancing the credibility of the analysis (25, 26). It may be limitations that the interview guide was not pilot tested, no repeat interviews were conducted, and that we did not return transcripts to the participants for comments and/or corrections nor used stakeholder check (26). Other potential limitations may include a lack of explicit triangulation of the findings through assessing other perspectives (for example service users) for knowing the implementation of Blend-A as well as the level of transferability of findings to other places in Europe and outside Europe.

### Conclusion

In conclusion, Blend-A was successful in its implementation process, but there were areas for improvement in the implementation process and in future projects as well. Familiarity and perceived normalcy of using Blend-A did not change significantly over time, but the cognitive attitude to Blend-A did. These findings may have important implications for the successful implementation and sustainability of Blend-A in the workplace. Overall, the therapists were optimistic about the possible use of a blended treatment format, and that this had a positive effect on the implementation process. Over time, the therapists developed confidence in benefits and disadvantages of a blended format.

While previous studies have not prioritized scrutinizing implementation processes; the present study shows that there is much to learn for management and implementers about improving and optimizing implementation processes for clinical studies.

## Data Availability

The raw data supporting the conclusions of this article will be made available by the authors, without undue reservation.
