# Supplementary material for: Therapist experiences with implementation of blended (iCBT and face-to-face) treatment of alcohol use disorder (Blend-A): mixed methods study
Source: Front Digit Health. 2025 Jun 17;7:1429582. doi: 10.3389/fdgth.2025.1429582 (PMC12209270; doi:10.3389/fdgth.2025.1429582)
Supplement: Supplementary file 1 [file Table1.docx]

| **Supplementary Material. Interview Guide** | |
| --- | --- |
| Introduction | Thank you for participating in this focus group interview. We would like to record it – is that okay? (if yes, start the record). The interview is going to be about how you have experienced Blend-A. The questions will be about your workday with Blend-A, Blend-A in your institution in relation to the patients, and user experiences with the platform. We will start with questions about your workday with Blend-A, and you are welcome to one at a time to tell and describe how you think and remember the experiences. |
| The workday with Blend-A | - How were you introduced to Blend-A? - How has it been for you to incorporate Blend-A as a part of your workday? - Are you and your colleagues confidential with using and offering Blend-A? - How have you remembered to use Blend-A at your workplace? - How have you been sharing experiences about Blend-A with your colleagues? - How do you and your colleagues use Blend-A (likewise or different)? - With method and cadence have you been choosing for conversation and written feedback – and how does it function? - Would you like to change something with the procedures you have around Blend-A at your workplace? If so, what and how? - How do you experience that your work is changed when you use Blend-A, in contrast to before when you didn´t have Blend-A?   Do you have more you would like to say about the work procedure with Blend-A? Otherwise, we proceed to Blend-A in the institution. |
| Blend-A in the institution | - What do you experience your institutions have gained through participating in the Blend-A project? - How does Blend-A fit in with your remaining treatment offers? - How will you assess that Blend-A have had an impact on your use of resources? - How does Blend-A fit into your institution´s needs and priorities?   Do you have mere you would like to say about Blend-A in relation to the institution? Otherwise, we proceed to questions about Blend-A and the patients. |
| Blend-A and the patients | - How have you experienced going from treating the patients face-to-face, to treating them online now? - Which group of patients will get Blend-A offered in your institutions (how do you assess if a patient gets Blend-A offered etc.)? - Have you experienced Blend-A making sense to the patient? In that case, how? - How have you used Blend-A with the patients (which model, creative use, waiting list, blended, only online and telephone, etc.)? - How has Blend-A had an impact on those patient courses you have had at your workplace (both in terms of content but also in terms of patients joining/maintaining, who otherwise would not have)?   Do you have more you would like to say in terms of the patients? Otherwise, we proceed to questions about user experiences with the internet-based treatment platform (the technical part of Blend-A functionality) |
| User experience with the internet-based treatment platform | - How did you get educated to use the platform? - How was your experience with using the platform in the beginning? - How was your experience when you have been using the platform for a while? - How is your experience with using it today? - How have you experienced the structure of the platform? - How have you experienced the content on the platform? - Have you experienced challenges with the use of the platform? If yes, which challenges? - Would you like to change something about the platform? If so, what and how? |
| Outro | Is there anything else you would like to say or any questions you would like to ask? Otherwise, we would like to thank you for participating in this interview and sharing your experiences. The interview will be anonymized and transcribed. Afterward, the interview and survey answers will be analyzed and presented as an evaluation of the project and articles around the implementation of Blend-A and the journey from treating face-to-face to treating digital. |
